# Temporal trends in concordance between ICD-coded and cardiac biomarker-classified hospitalisation rates for acute coronary syndromes: a linked hospital and biomarker data study

**DOI:** 10.1136/openhrt-2024-002995

**Published:** 2024-10-23

**Authors:** Dawit Zemedikun, Joseph Hung, Derrick Lopez, Matthew Knuiman, David Youens, Tom G Briffa, Frank Sanfilippo, Lee Nedkoff

**Affiliations:** 1 Cardiovascular Epidemiology Research Centre, School of Population and Global Health, The University of Western Australia, Perth, Western Australia, Australia; 2 Victor Chang Cardiac Research Institute, The University of Western Australia, Perth, Western Australia, Australia; 3 School of Medicine, The University of Western Australia, Perth, Western Australia, Australia; 4 School of Population Health, Curtin University, Perth, Western Australia, Australia

**Keywords:** Acute Coronary Syndrome, EPIDEMIOLOGY, Biomarkers

## Abstract

**Background:**

Since 2000, the definition of myocardial infarction (MI) has evolved with reliance on cardiac troponin (cTn) tests. The implications of this change on trends of acute coronary syndrome (ACS) subtypes obtained from routinely collected hospital morbidity data are unclear. Using person-linked hospitalisation data, we compared International Classification of Diseases (ICD)-coded data with biomarker-classified admission rates for ST-segment elevation MI (STEMI), non-STEMI (NSTEMI) and unstable angina (UA) in Western Australia (WA).

**Methods:**

We used linked hospitalisation data from all WA tertiary hospitals to identify patients with a principal diagnosis of STEMI, NSTEMI or UA between 2002 and 2016. Linked biomarker results were classified as ‘diagnostic’ for MI according to established criteria. We calculated age-standardised and sex-standardised rates (ASSRs) for ICD-coded versus biomarker-classified admissions by ACS subtypes and estimated annual change in admissions using Poisson regression adjusting for age and sex.

**Results:**

There were 37 272 ACS admissions in 30 683 patients (64.2% male), and 96% of cases had linked biomarker data, predominantly conventional cTn at the start and high-sensitive cTn from late 2013. Despite lower ASSRs, trends in MI classified with a diagnostic biomarker were concordant with ICD-coded admissions rates for both STEMI and NSTEMI. Between 2002 and 2010, STEMI rates declined by 4.1% (95% CI 5.0%, 3.1%) and 3.4% (95% CI 4.6%, 2.3%) in ICD-coded and biomarker-classified admissions, respectively, and both plateaued thereafter. For NSTEMI between 2002 and 2010, the ICD-coded and biomarker-classified rates increased 8.0% per year (95% CI 7.2%, 8.9%) and 8.0% (95% CI 7.0%, 9.0%), respectively, and both subsequently declined. For UA, both ICD-coded and biomarker-classified UA admission rates declined in a steady and concordant manner between 2002 and 2016.

**Conclusions:**

The present study supports the validity of using administrative data to monitor population trends in ACS subtypes as they appear to generally reflect the redefinition of MI in the troponin era.

**WHAT IS ALREADY KNOWN ON THIS TOPIC**

**WHAT THIS STUDY ADDS**

- By linking electronic hospitalisation data on patients with a principal diagnosis of STEMI, NSTEMI and UA with their cardiac biomarker results, we demonstrated that the rates and trends of ACS subtypes classified by administrative data and by biomarker results were concordant over the study period between 2002 and 2016.
- We confirmed that both administrative and biomarker-classified annual admission rates were falling for STEMI and rising for NSTEMI during the first half of the study period and thereafter plateaued or declined. In the case of UA, rates declined steadily over the whole study period.

**HOW THIS STUDY MIGHT AFFECT RESEARCH, PRACTICE OR POLICY**

- The present study supports the validity of using administrative data to monitor population trends in ACS subtypes as they appear to generally reflect the redefinition of MI in the troponin era.

## Introduction

 Acute coronary syndrome (ACS) is a major cause of death and morbidity worldwide.^1 2^ Over the past 20 years, there has been a significant change in the composition of ACS subtypes, with a marked decline in the incidence of ST-segment elevation myocardial infarction (STEMI) concomitant with a rise in non-STEMI (NSTEMI) and reciprocal fall in unstable angina (UA).^3^,^8^ These mixed trends are correlated with the the widespread adoption of increasingly sensitive cardiac troponin (cTn) immunoassays for detecting myocardial necrosis, and the redefinition of MI with increased emphasis on the role of troponin assays in identifying MI.^1 3 5 9 10^

Accurate monitoring of trends in ACS hospitalisations and its subtypes provides important information on coronary heart disease (CHD) patterns and the impact of primary and secondary coronary prevention at a population level. Routinely collected hospital administrative data allow surveillance of ACS trends if supported by periodic validation studies of disease coding as seen in previous studies using data from Western Australia (WA).^11^,^13^ The accuracy of hospitalisation data can be affected by changes in admission policies, case definition, and the sensitivity and specificity of diagnostic tests, particularly with advances in cTn immunoassays.^9 10^ By linking the results of cardiac biomarker tests to electronic hospital and emergency records, trends in ACS can be assessed according to biomarker status, enhancing the interpretation of administratively derived population-level ACS data and its subtypes.

Limited population-based studies have assessed the accuracy of ACS hospitalisations recorded in administrative data based on actual biomarker results.^312^,^14^ Since 2005, only one such validation study^13^ found that the sensitivity and specificity of the International Classification of Diseases, 10th revision, Australian Modification (ICD-10-AM) codes for MI subtypes in hospitalisation data were generally high based on reviews of ECGs and cardiac biomarkers in a sample of MI admissions in WA. However, this study relied on random samples of cases from selected study years and only up to 2013.^13^ Therefore, to determine the impact of MI redefinition in the troponin era on ACS hospitalisation data over recent decades, this study assessed and compared temporal trends in ACS subtypes using ICD-10-AM coded hospitalisation rates and cardiac biomarker-classified ACS hospitalisation rates in WA between 2002 and 2016.

## Methods

### Data sources

This study used data from the WA Department of Health’s Hospital Morbidity Data Collection (HMDC) and Emergency Department Data Collection (EDDC), with linkage through the WA Data Linkage System.^15^ The HMDC study dataset comprised all CHD hospitalisations in WA from 1985 to 2017 and included demographic variables, principal and additional diagnosis fields, in-hospital procedures, hospital type and admission status. The EDDC dataset comprised all emergency department (ED) presentations for people with a CHD hospitalisation record from 2002 to 2017. Results of cardiac biomarker testing undertaken in hospital or ED were obtained from the main public pathology provider (Pathwest) for tertiary hospital admissions in the HMDC study dataset during the study period. Cardiac biomarker test results included creatine kinase (CK), conventional cTn and high-sensitivity cTn (hs-cTn). The upper limit of normal (ULN) values for each assay and date of collection were available.

### Cohort identification

The study cohort included all hospitalisations in individuals aged ≥20 years admitted to a WA tertiary hospital who were also Greater Perth metropolitan area residents, with a principal discharge diagnosis of STEMI (ICD-10-AM I21.0-I21.3), NSTEMI (I21.4) or UA (I20.0) from 1 January 2002 to 31 December 2016 (online supplemental appendix 1). The estimated resident population of WA was 2.5 million in 2016 with about 78% residents in metropolitan Perth.^16^ A series of linked hospitalisations were considered part of an episode of care following our previously published methods.^12^ The admission date/time for the episode in this case was the admission date/time of the first hospitalisation in the sequence. Because more than one ACS subtype can be recorded as the principal diagnosis within a linked admission, a diagnosis hierarchy was used (STEMI>NSTEMI>UA).^12^ We did not report separately on admissions coded as unspecified MI (I21.9), which comprise only <5% of total MI admissions, but these were included in the ‘all MI’ category. A patient could contribute to multiple ACS hospitalisations and different ACS subtype events during the study period.

### Ascertainment of cardiac biomarker status and classification of ACS

Cardiac biomarker results were linked to each hospital admission based on laboratory collection date and time relative to the ED presentation date or hospital admission date/time (online supplemental appendix 2). Between 2006 and 2008, there was a ~20% reduction in biomarker data able to be linked to the admissions (online supplemental appendix 3), hence we did not undertake biomarker classification for these study years. The reason for the reduction was likely to be due to pathology data being stored in a separate data system and not provided to the research team, which was beyond the control of the researchers. Admissions with any linked biomarker result available (biomarker classifiable) and admissions with cTn or hs-cTn available (cTn/hs-cTn classifiable) were identified. The cTn/hs-cTn results took precedence over CK and were considered diagnostic where there were ≥2 tests available, with at least one value >99th percentile of the ULN and at least 20% rise or fall on serial results (diagnostic biomarkers, online supplemental appendix 4).^10^ The median time between the initial and peak measurement was about 4 hours for cTn-I and 8 hours for cTn-T assay types. Sex-specific 99th percentile thresholds were applied for hs-cTn assays. Where only CK results were available, a diagnostic biomarker required at least one test during the admission which was >2× the ULN for that assay. Biomarker-classified STEMI and NSTEMI were those that met the diagnostic biomarker criteria; UA cases were those with non-diagnostic biomarkers. We also conducted a sensitivity analysis where we included equivocal classifications (online supplemental appendix 4) as diagnostic for STEMI and NSTEMI.

### Patient characteristics

Age, sex, length of stay, admission status and hospital type were obtained from the hospitalisation records. Comorbidities were identified using all 21 available diagnosis fields from hospitalisations up to 20 years prior to the first ACS hospitalisation in the study period and for each of the subtypes separately (online supplemental appendix 5).

### Statistical analysis

Categorical data are reported as frequencies and percentages and continuous data as means and SDs. Hospitalisation rates for each ACS subtype were measured for ICD-coded, biomarker-classifiable and cTn/hs-cTn-classifiable admissions, and for admissions with diagnostic biomarkers and diagnostic cTn/hs-cTn (for those with troponin results available). UA rates were calculated for cases with non-diagnostic biomarkers and non-diagnostic cTn/hs-cTn. Annual age-standardised and sex-standardised rates (ASSRs) were calculated by the direct method and standardised using 5-year age groups, with the 2016 Australian population as the standard. Denominators were the annual population numbers for the Greater Perth region obtained from the Australian Bureau of Statistics.^16^ Age-standardised rates were also calculated stratified by sex and age group. Rate ratios (RRs) were calculated by comparing diagnostic biomarker rates and admissions with the equivalent biomarker-classifiable groups. We estimated average annual percentage changes in hospitalisation rates using Poisson regression models adjusting for 5-year age group and sex. Because of the non-linear trends in STEMI and NSTEMI rates, we fitted separate models for two time periods 2002–2010 and 2011–2016. All analyses were performed by using Stata V.17.0SE (StataCorp) and SAS V.9.4 (SAS Institute).

## Results

### Cohort characteristics

Between 2002 and 2016, a total of 37 272 ACS hospitalisations representing 30 683 patients (64.2% male) were identified. Mean age was lower for STEMI than NSTEMI cases (men 61.6 years vs 68.0 years; women 71.5 years vs 75.6 years) (table 1). The prevalence of prior CHD and other major comorbidities was consistently lower for patients with STEMI compared with NSTEMI in both sexes. Mean age of UA cases was 66.3 years in men and 72.3 years in women, and a higher proportion had a prior history of CHD compared with STEMI and NSTEMI cases in both sexes.

**Table 1:** Baseline characteristics and comorbidities in the cohort

Patients (n)	Male			Female		
	STEMI (n=5252)	NSTEMI (n=8423)	UA (n=6118)	STEMI (n=1948)	NSTEMI (n=4957)	UA (n=3988)
All admissions, n (%)	5503 (23.0)	9856 (41.2)	8571 (35.8)	2020 (15.1)	5926 (44.4)	5396 (40.4)
Average annual admissions, n	459	821	714	168	494	450
Mean age, years (SD)	61.6 (13.4)	68.0 (13.9)	66.3 (12.6)	71.5 (14.6)	75.6 (14.0)	72.3 (13.0)
Mean LOS, days (SD)	5.2 (8.8)	7.5 (47.4)	4.2 (8.8)	7.4 (12.7)	8.6 (14.4)	4.4 (8.1)
Admission status, n (%)						
Emergency	5281 (96.0)	9549 (96.9)	7447 (86.9)	1953 (96.7)	5812 (98.1)	4926 (91.3)
Comorbidities*, n (%)						
Prior CHD	1135 (21.6)	3768 (44.7)	4190 (68.5)	527 (27.1)	2276 (45.9)	2536 (63.6)
Prior MI	628 (12.0)	1825 (21.7)	2242 (36.7)	287 (14.7)	1114 (22.5)	1189 (29.8)
Heart failure	771 (14.7)	2200 (26.1)	1197 (19.6)	523 (26.9)	1955 (39.4)	1036 (26.0)
Atrial fibrillation	667 (12.7)	1827 (21.7)	1294 (21.2)	366 (18.7)	1356 (27.4)	909 (22.8)
Valvular heart disease	248 (4.7)	879 (10.4)	576 (9.4)	158 (8.1)	721 (14.6)	529 (13.3)
Hypertension	2575 (49.0)	5804 (68.9)	4595 (75.1)	1261 (64.7)	3894 (78.6)	3153 (79.1)
Peripheral vascular disease	358 (6.8)	1308 (15.5)	922 (15.1)	223 (11.5)	774 (15.6)	595 (14.9)
Stroke	224 (4.3)	627 (7.4)	403 (6.6)	153 (7.9)	480 (9.7)	287 (7.2)
COPD	376 (7.2)	1112 (13.2)	873 (14.3)	272 (14.0)	933 (18.8)	818 (20.5)
Chronic kidney disease	496 (9.4)	1960 (23.3)	1151 (18.8)	303 (15.6)	1333 (26.9)	807 (20.2)
Diabetes mellitus	1084 (20.6)	2675 (31.8)	1932 (31.6)	531 (27.3)	1668 (33.7)	1260 (31.6)
Alcohol and drug history	484 (9.2)	936 (11.1)	728 (11.9)	124 (6.4)	313 (6.3)	251 (6.3)
Mental health	391 (7.4)	885 (10.5)	816 (13.3)	275 (14.1)	868 (17.5)	772 (19.4)

* Comorbidities calculated at patient level, and based on the comorbidity status of the patient at the time of the first admission for that subtype during the study period.

CHD, coronary heart diseaseCOPDchronic obstructive pulmonary diseaseLOS, length of stay; MImyocardial infarctionNSTEMI, non-STEMI; STEMI, ST-elevation MI; UA, unstable angina

### Biomarker usage and changes over time

**Figure 1:**
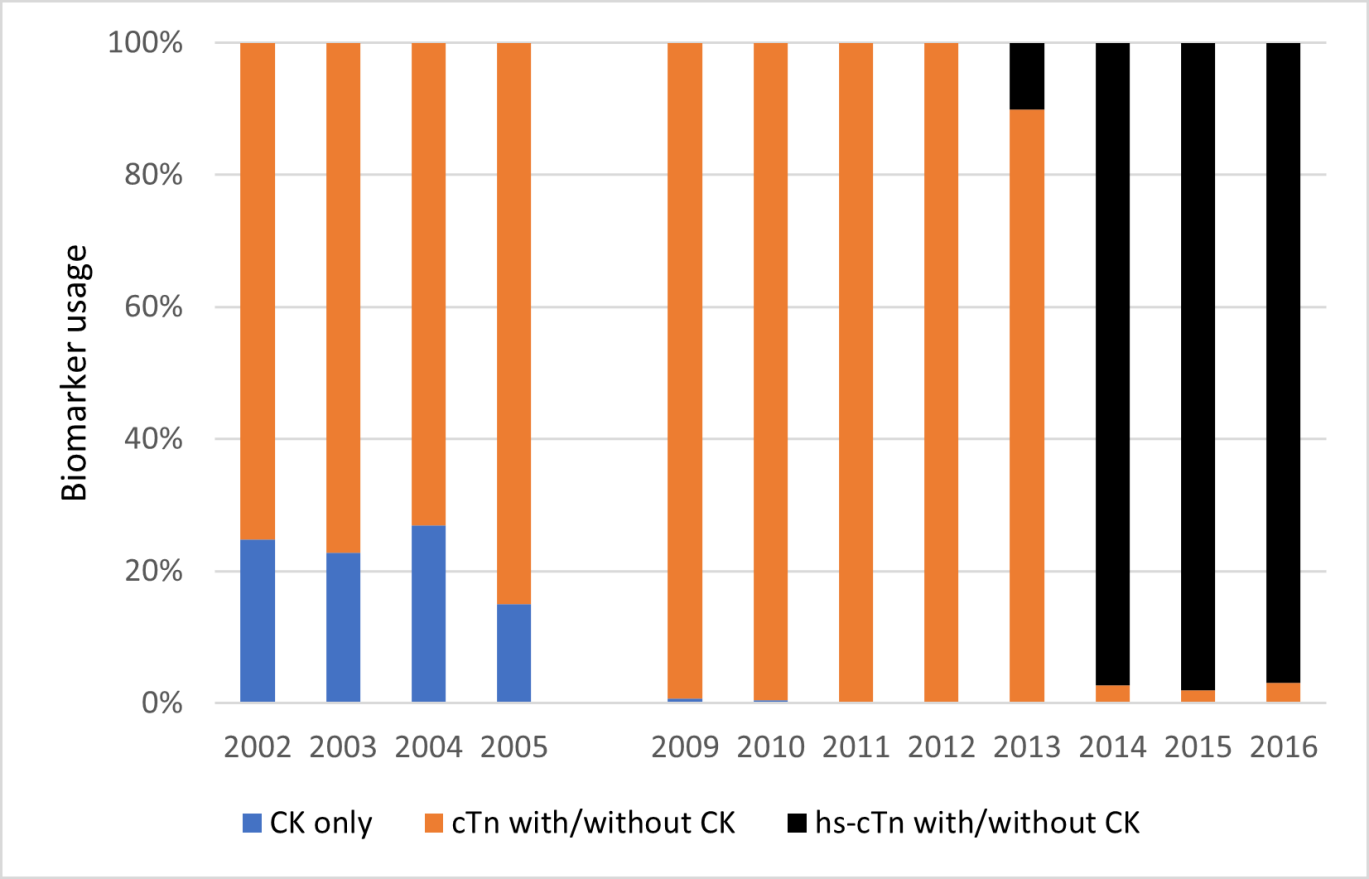
Temporal changes in type of cardiac biomarker used for classification of acute coronary syndrome admissions during the study period. CK, creatine kinase; hs-cTn, high-sensitivity cardiac troponin.

Conventional cTn or hs-cTn was the predominant biomarker in use overall while the use of ‘CK only’ was restricted to 2002–2005 and constituted <25% of the annual ACS admissions. In late 2013, hs-cTn was introduced and its utilisation rapidly increased to around 97% of all ACS admissions by 2016 (figure 1). Excepting 2006–2008 years, linked biomarker data were available for 97.1% and 98.0% of ICD-coded STEMI and NSTEMI cases, respectively, with little change during the study period (table 2). Of those with any biomarker test available, 87.1% of STEMI and 82.4% of NSTEMI ICD-coded cases had a diagnostic biomarker. When the analysis was restricted to cTn/hs-cTn tests, the annual proportions and trend of diagnostic cTn/hs-Tn for STEMI and NSTEMI were similar (table 2). Among ICD-coded UA cases with an available biomarker, 79.6% and 77.3% were classified as having a non-diagnostic biomarker based on any biomarker and cTn/hs-Tn test results, respectively, with no discernible trends over time (table 2).

**Table 2:** Number and proportion of ICD-coded ACS admissions according to cardiac biomarker use and classification

Year	ICD-coded, n	Test performed, n (%)*		ACS classification, n (%)	
		Biomarker-classifiable	cTn/hs-cTn-classifiable	Diagnostic biomarker†	Diagnostic cTn/hs-cTn‡
STEMI					
2002	748	710 (94.9)	528 (70.6)	619 (87.2)	489 (92.6)
2003	627	592 (94.4)	458 (73.0)	509 (86.0)	410 (89.5)
2004	604	559 (92.5)	410 (67.9)	479 (85.7)	372 (90.7)
2005	563	536 (95.2)	463 (82.2)	449 (83.8)	400 (86.4)
2009	630	591 (93.8)	588 (93.3)	503 (85.1)	502 (85.4)
2010	636	630 (99.1)	629 (98.9)	552 (87.6)	551 (87.6)
2011	583	576 (98.8)	575 (98.6)	504 (87.5)	504 (87.7)
2012	604	602 (99.7)	602 (99.7)	529 (87.9)	529 (87.9)
2013	573	570 (99.5)	570 (99.5)	534 (93.7)	534 (93.7)
2014	648	640 (98.8)	640 (98.8)	581 (90.8)	581 (90.8)
2015	608	602 (99.0)	602 (99.0)	533 (88.5)	533 (88.5)
2016	699	696 (99.6)	696 (99.6)	571 (82.0)	571 (82.0)
Overall	7523	7304 (97.1)	6761 (89.9)	6363 (87.1)	5976 (88.4)
NSTEMI					
2002	618	591 (95.6)	508 (82.2)	495 (83.8)	455 (89.6)
2003	713	692 (97.1)	553 (77.6)	579 (83.7)	493 (89.2)
2004	854	813 (95.2)	651 (76.2)	641 (78.8)	567 (87.1)
2005	932	887 (95.2)	780 (83.7)	696 (78.5)	647 (82.9)
2009	1474	1406 (95.4)	1395 (94.6)	1104 (78.5)	1103 (79.1)
2010	1540	1513 (98.2)	1511 (98.1)	1193 (78.8)	1193 (79.0)
2011	1809	1790 (98.9)	1788 (98.8)	1417 (79.2)	1417 (79.3)
2012	1744	1722 (98.7)	1722 (98.7)	1404 (81.5)	1404 (81.5)
2013	1573	1559 (99.1)	1556 (98.9)	1355 (86.9)	1355 (87.1)
2014	1492	1487 (99.7)	1486 (99.6)	1295 (87.1)	1295 (87.1)
2015	1604	1592 (99.3)	1592 (99.3)	1374 (86.3)	1374 (86.3)
2016	1429	1411 (98.7)	1411 (98.7)	1176 (83.3)	1176 (83.3)
Overall	15 782	15 463 (98.0)	14 953 (94.7)	12 739 (82.4)	12 479 (83.5)
UA				Non-diagnostic biomarker†	Non-diagnostic cTn/hs-cTn‡
2002	1850	1662 (89.8)	1192 (64.4)	1355 (81.5)	906 (76.0)
2003	1644	1528 (92.9)	1159 (70.5)	1216 (79.6)	864 (74.5)
2004	1675	1532 (91.5)	1063 (63.5)	1300 (84.9)	842 (79.2)
2005	1521	1400 (92.0)	1158 (76.1)	1163 (83.1)	930 (80.3)
2009	1080	1007 (93.2)	1002 (92.8)	766 (76.1)	761 (75.9)
2010	1243	1193 (96.0)	1183 (95.2)	915 (76.7)	905 (76.5)
2011	1149	1088 (94.7)	1086 (94.5)	860 (79.0)	858 (79.0)
2012	1029	990 (96.2)	985 (95.7)	761 (76.9)	756 (76.8)
2013	737	701 (95.1)	699 (94.8)	520 (74.2)	518 (74.1)
2014	755	716 (94.8)	714 (94.6)	546 (76.3)	544 (76.2)
2015	617	583 (94.5)	581 (94.2)	465 (79.8)	463 (79.7)
2016	667	602 (90.3)	600 (90.0)	480 (79.7)	478 (79.7)
Overall	13 967	13 002 (93.1)	11 422 (81.8)	10 347 (79.6)	8825 (77.3)

* Represents which biomarker test was used for classification of an ACS admission. Biomarker-classifiable includes admissions where cTn/hs-cTn results (±CK results) or only CK results were available. cTn/hs-cTn-classifiable includes admissions where any troponin assay was available for classification. Both are calculated as a percentage of ICD-coded admissions.

† Calculated as a percentage of biomarker-classifiable admissions. When both CK and cTn/hs-cTn results were available on the same admission, the classification was based on the cTn or hs-cTn result.

‡ Calculated as a percentage of cTn/hs-cTn-classifiable.

ACSacute coronary syndromeCKcreatine kinasecTncardiac troponinhs-cTNhigh-sensitivity cTnICDInternational Classification of DiseasesNSTEMInon-STEMISTEMIST-elevation myocardial infarctionUAunstable angina

### Temporal trends in ASSRs

**Figure 2:**
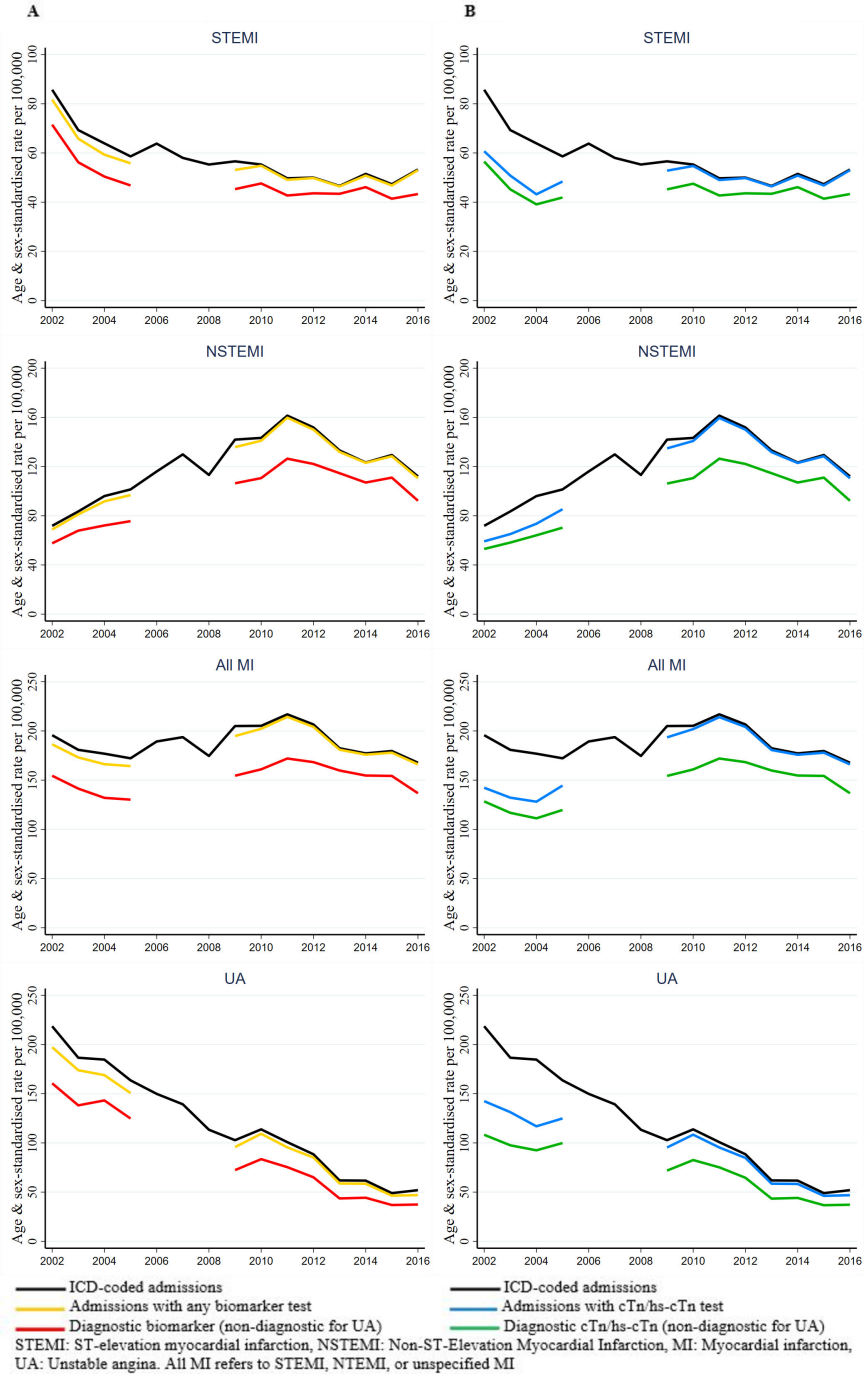
Age-standardised and sex-standardised admission rates stratified by biomarker classification status in acute coronary syndrome subtypes. Comparisons with ICD-coding with either (A) any biomarker test or elevated biomarker test result and (B) any troponin assay test or elevated troponin test result. ICD, International Classification of Diseases.

The annual biomarker-classifiable admission ASSRs were similar to ICD-coded admission rates across all ACS subtypes (figure 2A). There was a similar pattern for cTn/hs-Tn-classifiable rates except between 2002 and 2005 when ~25% had only CK results available (figure 2B). The diagnostic biomarker-classified rates (or non-diagnostic biomarker for UA) were lower but concordant than the equivalent biomarker-classifiable rates for each ACS subtype (including all MIs) (figure 2A). The overall RR comparing admission rates of diagnostic (STEMI/NSTEMI/all MIs)) or non-diagnostic biomarkers (UA) versus biomarker-classifiable rates were 0.87, 0.82, 0.83 and 0.79 for STEMI, NSTEMI, all MIs and UA, respectively, with no consistent change over the period (table 3). The overall pattern of results was very similar when restricted to ACS admissions with cTn/hs-Tn only (figure 2B and table 3). When cases with an equivocal classification were included in the diagnostic biomarker groups, there was a closer approximation of STEMI and NSTEMI diagnostic biomarker-classified rates compared with biomarker-classifiable admission rates, and trends were largely consistent with the main analysis (online supplemental appendix 6). No major differences in patterns by sex were observed in the stratified analyses (online supplemental appendices 7,8).

**Table 3:** Age-standardised rate ratios comparing diagnostic biomarker rates with biomarker-classifiable rates by acute coronary syndrome subtype

	Biomarker-classified*				cTn/hs-cTn-classified†			
**Year**	**STEMI**	**NSTEMI**	**All MIs**	**UA**	**STEMI**	**NSTEMI**	**All MIs**	**UA**
2002	0.88	0.84	0.83	0.81	0.93	0.90	0.90	0.76
2003	0.85	0.84	0.82	0.79	0.89	0.89	0.88	0.74
2004	0.85	0.79	0.79	0.85	0.91	0.87	0.87	0.79
2005	0.84	0.78	0.79	0.83	0.87	0.82	0.83	0.80
2009	0.85	0.78	0.79	0.76	0.86	0.79	0.80	0.75
2010	0.87	0.78	0.80	0.76	0.87	0.79	0.80	0.76
2011	0.87	0.79	0.80	0.79	0.87	0.79	0.80	0.79
2012	0.88	0.81	0.82	0.76	0.88	0.81	0.82	0.76
2013	0.94	0.87	0.88	0.74	0.94	0.87	0.88	0.74
2014	0.91	0.87	0.88	0.76	0.91	0.87	0.88	0.76
2015	0.88	0.86	0.87	0.80	0.88	0.86	0.87	0.79
2016	0.82	0.83	0.82	0.80	0.82	0.83	0.82	0.79
Overall	0.87	0.82	0.83	0.79	0.88	0.84	0.85	0.77

All MIs include STEMI, NSTEMI and unspecified MI.

* Ratio of diagnostic rates with biomarker-classifiable rates. For UA, the comparison was between non-diagnostic biomarker versus biomarker-classifiable rates.

† Ratio of diagnostic cTn/hs-cTn rates with cTn/hs-cTn-classifiable rates. For UA, the comparison was between non-diagnostic cTn/hs-cTn vs cTn/hs-cTn-classifiable rates.

hs-cTnhigh-sensitivity cardiac troponinMImyocardial infarctionNSTEMInon-STEMISTEMIST-elevation MIUAunstable angina

ASSRs of ICD-coded STEMI rates declined in the first half of the study period (2002–2010) and thereafter plateaued, while NSTEMI and all MI rates increased up to 2010 and declined in the years subsequent to 2012 (figure 2). For ICD-coded UA admissions, there was a steady decline in rates throughout the entire study period. In the overall WA population, similar trends were evident for ICD-coded admission rates for ACS subtypes and when stratified by sex, except that NSTEMI and all MI rates plateaued after peaking between 2010–2012 (online supplemental appendix 9).

### Average annual percentage changes in ACS Hospitalisation

Table 4 shows the age-adjusted and sex-adjusted average annual percentage change in ACS subtype rates, stratified by biomarker classification status. During (2002–2010), ICD-coded STEMI rates declined by 4.1%/year (95% CI 5.0%, 3.1%) and diagnostic-biomarker rates by 3.4% (95% CI 4.6%, 2.3%), with no significant changes after 2010. There was no change in diagnostic cTn/hsTn-classified STEMI rates between 2002 and 2010. For NSTEMI, both the ICD-coded and diagnostic biomarker rates increased by 8.0%/year between 2002 and 2010, and similar annual increments were seen in diagnostic cTn/hs-Tn NSTEMI rates. Thereafter, NSTEMI rates declined significantly across all classifications. The increase in all MI rates in the earlier time period reflected the opposing trends between STEMI and NSTEMI, with significant declines in the later period. UA rates declined significantly across all classifications in both periods. Age-specific and sex-specific analyses showed a generally similar direction of trends between ICD-coded and diagnostic biomarker rates although with some differences in magnitude of change, and with generally greater concordance in the later period (online supplemental appendix 10).

**Table 4:** Average annual percentage changes in admission rates for 2002–2010 and 2011–2016 by classification status in acute coronary syndrome subtypes

Average annual % change (95% CI)		
	2002–2010*	**2011–2016**
STEMI		
ICD coded	−4.1 (−5.0, 3.1)	0.7 (−1.1, 2.7)
Diagnostic biomarker	−3.4 (−4.6, 2.3)	−0.2 (−2.2, 1.8)
Diagnostic cTn/hs-cTn	0.0 (−1.2, 1.3)	−0.2 (−2.2, 1.8)
NSTEMI		
ICD coded	8.0 (7.2, 8.9)	−6.7 (−7.8, 5.6)
Diagnostic biomarker	8.0 (7.0, 9.0)	−5.4 (−6.6, 4.2)
Diagnostic cTn/hs-cTn	9.9 (8.8, 10.9)	−5.4 (−6.6, 4.2)
All MI		
ICD coded	1.3 (0.7, 1.9)	−4.9 (−5.9, 4.0)
Diagnostic biomarker	1.6 (0.9, 2.3)	−4.1 (−5.1, 3.0)
Diagnostic cTn/hs-cTn	4.4 (3.7, 5.2)	−4.1 (−5.1, 3.0)
UA		
ICD coded	−8.7 (−9.3, 8.1)	−14.1 (−15.5, 12.7)
Non-diagnostic biomarker	−8.8 (−9.6, 8.0)	−14.5 (−16.2, 12.8)
Non-diagnostic cTn/hs-cTn	−3.7 (−4.5, 2.8)	−14.5 (−16.2, 12.8)

All MIs include STEMI, NSTEMI and unspecified MI.

* Excludes 2006–2008 for non-ICD-coded trends.

hs-cTnhigh-sensitivity cardiac troponinICDInternational Classification of DiseasesMImyocardial infarctionNSTEMInon-STEMISTEMIST-elevation MIUAunstable angina

## Discussion

This study uniquely investigated temporal concordance between linked ACS subtype hospitalisation data based on ICD coding and those classified using cardiac biomarker thresholds. Initially, the cardiac biomarker used was predominantly conventional cTn (with or without CK), with rapid adoption of hs-cTn testing from late 2013. While biomarker-classifiable admission rates for each ACS subtype were around 13%–20% higher than the rates where the biomarker results were diagnostic, this disparity remained reasonably consistent throughout the study period. Age-adjusted and sex-adjusted trends in admission rates of both ICD-coded and biomarker-classified ACS subtypes were concordant, with rates of STEMI falling, concurrent with rising rates of NSTEMI in the first half of the study period while UA rates declined steadily over the whole period. These findings suggest that ACS trends using ICD-coded hospitalisation data align with rates based on diagnostic biomarkers across each of the ACS subtypes.

### Concordance of ICD-coded and biomarker-classified ACS rates by subtype

Limited studies have compared temporal trends in ICD-coded and biomarker-classified ACS hospitalisations.^3 12 14 17^ These studies were limited by smaller sample sizes and were mostly undertaken before the redefinition of MI in 2000^18^ and focused on MI overall rather than subtypes. The studies generally found that the changing diagnostic criteria for MI posed challenges in assessing population-level MI trends.^14 17^ For instance, a WA study found a 17% increase in MI rates in 1998 vs 2003 based on American Heart Association criteria, compared with a 3.5% decline in ICD-coded rates.^17^ This discrepancy was attributed to a high proportion of false-negative ICD-coded cases relative to troponin-based diagnosis, likely due to reluctance by clinicians to diagnose MI based on relatively small increases in troponin levels at that time.^12^ Similarly, a US study reported a 20% decline in MI incidence from 1987 to 2006 using CK/CK-MB criteria, while no change was seen with troponin-based criteria.^3^ A more recent validation study by our group showed generally high sensitivity and specificity of ICD-coded STEMI and NSTEMI hospitalisations based on ECG and cardiac biomarker results between 2003 and 2013.^13^

In the current study, the fact that the ICD-coded ACS rates for all subtypes were generally higher than diagnostic biomarker-classified rates is likely because clinical diagnosis is based also on presenting symptoms and ECG criteria and not solely on biomarkers, especially for STEMI. There is also recognition that elevated cTn levels can occur due to chronic myocardial injury unrelated to plaque disruption, and ICD-coding does not yet accommodate the coding of type 1 or 2 classification.^9 10^ Nevertheless, the concordance between ICD-coded and diagnostic biomarker ACS rates suggests improved consistency in the recording of ACS subtypes in administrative datasets.

### Temporal trends in ICD-coded and biomarker-classified ACS subtypes

The introduction of troponin testing was expected to increase MI incidence and shift the spectrum of ACS from more severe forms (STEMI) to less severe forms (NSTEMI) while reducing UA incidence.^1 9 10^ Epidemiological studies up to the mid to late 2000s demonstrated this shift.^13^,^8^ Our study, covering the era of hs-Tn testing from late 2013, confirmed falling ICD-coded and diagnostic biomarker STEMI rates and rising NSTEMI rates up to 2010, with attenuation of trends thereafter. UA admission rates declined significantly throughout the study across all classifications continuing with the introduction of hs-cTn.^8 10 19^ A randomised controlled trial demonstrated that the introduction of hs-cTn testing disproportionately increased type 2 MI cases and myocardial injury diagnosis compared with type 1 MI.^20^ Despite this, the concordance between ICD-coded and diagnostic biomarker-classified rates remained consistent following the introduction of hs-cTn in our jurisdiction, suggesting that trends from administrative data in post-hs-cTn introduction align with clinical definitions.

Few studies have examined the diagnostic accuracy of recording of UA in linked hospitalisation data. The changes in MI diagnostic criteria with use of increasingly sensitive cTn assays have impacted UA diagnosis, leading to declining UA hospitalisation rates.^21^,^23^ Epidemiological data on UA are less robust than for MI because UA diagnosis is based on symptoms of acute myocardial ischaemia with or without ECG changes and in the absence of diagnostic biomarkers.^24^ Over 20% of patients initially diagnosed with UA have no significant coronary artery disease on angiography.^25^ Our results indicate that ICD-coded UA rates generally overestimated non-diagnostic biomarker rates by ~20%, however, there was good concordance between the two classifications over the study period. The shift in the makeup of ACS may also be influenced by factors other than troponin testing. The decline in STEMI rates preceded widespread adoption of cTn testing,^1 3 4^ with increased use of cardioprotective medications in primary and secondary prevention associated with considerable reductions in STEMI risk due to their plaque-stabilising and antithrombotic effects.^1 2^

### Strengths and limitations

The study limitations were first that records from 2006 to 2008 were excluded due to substantially lower rates of biomarker data. The exclusion was important to avoid potential bias in concordance and trend measures, as estimates for those years would have been significantly underestimated. The use of Poisson regression models excluding these years ensured a linear trend, rather than modelling significant rate reductions during this period. Only hospitalisations for Perth metropolitan residents admitted to WA tertiary hospitals were included, as rural and non-tertiary metropolitan hospital pathology data were unavailable. However, the validity of rates was ensured by careful selection of population-level denominators and using data from the main public pathology provider for WA (PathWest).

While the ICD-10 coding available in Australia does not enable differentiation between types 1 and 2 MIs, this is unlikely to have impacted our main results. The MI cases now considered type 2 cases would likely still have been captured in ICD-coded data, although lacking information on the underlying pathophysiology of the event. We could not determine if changes in troponin assay sensitivity impacted rates or trends in ACS subtypes at specific time points since assay changes with different ULNs occurred at various times across different hospitals. Rates and trends for each ACS subtype using whole-population WA data may not be reflective of overall rates and trends in our state as they are restricted to tertiary hospital admissions in metropolitan residents only and are shown to provide context to our study findings. Study strengths include the population-based nature of our study with a large sample size, allowing for examination of annual rates and trends in each ACS subtype, and age-specific and sex-specific concordance. Robust methods reduced the inflation of hospitalisation rates due to interhospital transfers^26^ and ensured accurate linkage of pathology data to the hospital dataset.

## Conclusion

This large, population-based study demonstrated that biomarker-classified ACS trends are largely concordant with ICD-coded trends for each ACS subtype. Despite shifts from CK to conventional cTn and then hs-cTn assays during our study period, the present study highlights the validity of using linked hospitalisation data to monitor long-term trends in ACS since adoption of the Universal Definition of MI. Despite changing cardiac biomarkers and assay sensitivity, these findings reaffirm the continued use of linked hospitalisation data for monitoring ACS and its subtypes as important measures of CHD burden and for demonstrating the impact of coronary prevention at a population level. However, the relatively high rates of patients diagnosed with NSTEMI in the troponin era have implications for clinical and ongoing management and prognosis. Periodic validation of administrative data is also needed to ensure the approach remains fit for purpose.

## supplementary material

10.1136/openhrt-2024-002995 online supplemental file 1

## Data Availability

Data may be obtained from a third party and are not publicly available.
