# Supplementary material for: Temporal trends in concordance between ICD-coded and cardiac biomarker-classified hospitalisation rates for acute coronary syndromes: a linked hospital and biomarker data study
Source: Open Heart. 2024 Oct 23;11(2):e002995. doi: 10.1136/openhrt-2024-002995 (PMC11499754; doi:10.1136/openhrt-2024-002995)
Supplement: online supplemental file 1 [file openhrt-11-2-s001.pdf]

Supplementary materials

Appendix 1: Methods for assigning location of residence status to ACS cases

The HMDC dataset contained several variables that could be used as a proxy for assigning residence status. These included postcode, Statistical Areas Level 2 (SA2) codes for 2011, radius, Statistical Local Area (SLA) codes for census years 1996, 2001, and 2006, Local Government Area (LGA) codes for census years 1996, 2001, 2006, and 2011, and various Socio-Economic Indexes for Areas (SEIFA) variables. Of these, SA2 codes and postcode were found to be the most relevant to map or compare against the denominator data. There was no missingness in postcode data while SA2 codes had 2,986 (1.7%) records missing in the initial tertiary hospital cohort which included patients living in rural areas or other states but admitted to hospitals in the Greater Perth area. Two external datasets were used to merge and assign the residential area names to the SA2 codes in the cohort.

1 – SA2 Australian Statistical Geography Standard (ASGS) Edition 2011 data: This was a dataset from the Australian Bureau of Statistics (ABS) which include SA2 and higher geographical codes and names and is available at https://www.abs.gov.au/AUSSTATS/abs@.nsf/DetailsPage/1270.0.55.001July%202011?OpenDocument. This data was merged by SA2 codes to assign location of residence and it matched 100% of SA2 records in the cohort. The 2011 edition was selected as it provided the best match for the SA2 data in our cohort. SA2s from ASGS Edition 2016 and ASGS Edition 2021 were tested but there were mismatches for both versions due to some changes in SA2 codes in the later census years.

2 - Postcode 2011 to Statistical Area Level 2 2011 data: This was a correspondence dataset from ABS for mapping postcode to SA2 and is available at https://www.abs.gov.au/AUSSTATS/abs@.nsf/DetailsPage/1270.0.55.006July%202011?OpenDo

cument. It was used to fill the gaps where SA2 code data were missing in the cohort. A single postcode could fall into more than one SA2s, hence a ratio and % were available indicating the proportion of the postcode covering each SA2 area. The dataset was initially processed to keep a single SA2 with the highest ratio or % of coverage for each postcode. This was then merged by postcode to the cohort matching 1,523 (51.0%) of the records missing SA2 codes. The remaining postcodes with missing SA2 were manually checked and were all excluded for being non-metropolitan area (Greater Perth) postcodes. We further reduced our cohort to those records that met the ABS definition of Greater Perth by SA2. The city of Mandurah was excluded from our metropolitan definition of the numerator for the whole of the study period as it was not included in the existing denominator data.

Appendix 2: Flowchart showing linkage of ED and hospital records to biomarker data for classification of results

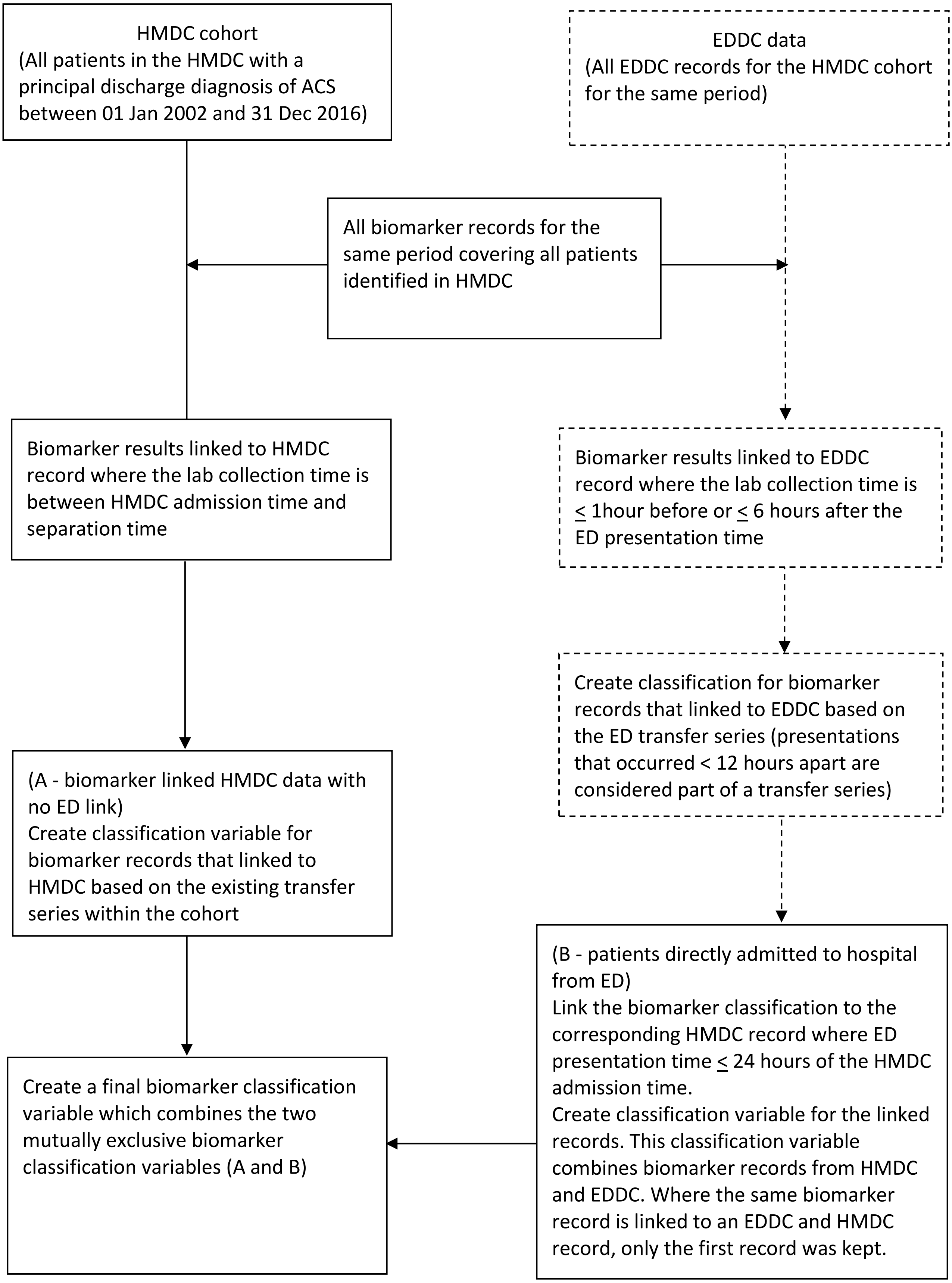

Appendix 3: Biomarker data linkage rate in the available data showing a reduction in biomarker records between 2006-2008

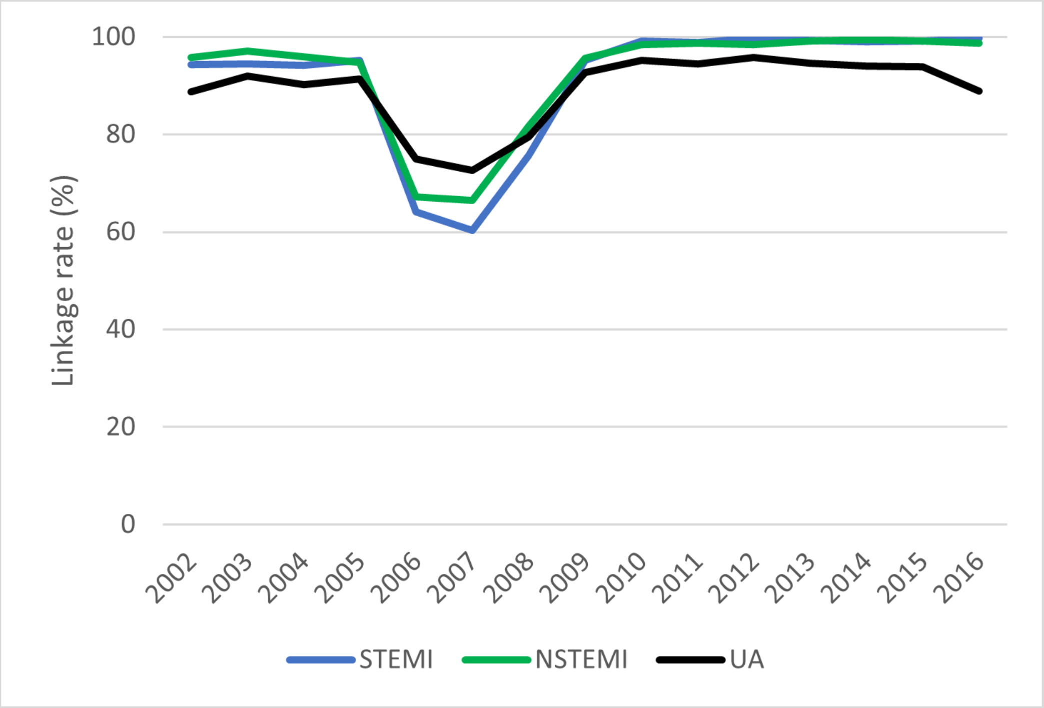

Appendix 4: Cardiac biomarker classification for an episode of care

**Criteria***	**Classification**
STEMI/NSTEMI	
≥2 troponin tests with acute rise or fall relative to maximum troponin value (largest difference from the maximum troponin result is >20%), and at least 1 troponin value >99th percentile),<br>OR<br>One or more CK value > 2xULN for the assay	Diagnostic
Single elevated troponin level (>99th percentile for the assay)<br>OR<br>One or more CK results are >ULN but <2xULN for that assay (with no diagnostic CK result present)	Equivocal
≥ 2 troponin tests without a significant rise or fall (largest difference between troponin values and max value is ≤ 20%), and at least 1 troponin value is >99th percentile.	Chronic**
Single troponin test which is ≤99th percentile for the assay<br>OR<br>All CK values at or below ULN for the assay	Normal

*Where both troponin and CK results were available for the same admission, the classification for that admission was based on the cTn or hs-cTn results.
**In the absence of diagnostic serial results during the same admission.

Appendix 5: List of ICD codes used for identification of the cohort and comorbidities

**Comorbidity**	**ICD-9/ICD9-CM** (01 Jan 1988 - 30 Jun 1999)	**ICD-10-AM** (01 Jul 1999 – 31 Dec 2016)
STEMI	410.0-410.6, 410.8	I21.0-I21.3
NSTEMI	410.7	I21.4
Unspecified MI	410.9	I21.9
Unstable Angina	411.1	I20.0
Prior (CHD)	410-414	I20-I25
Prior MI	410	I21, I22
Heart failure	428	I50, U82.2
Valvular heart disease (VHD)	393-398, 421.0, 421.1, 421.9, 424.0, 424.1, 424.2, 424.3, 424.9	I05-I09, I30.9, I34-I39
Hypertension	401-405	I10-I15, U82.3
Peripheral vascular disease (PVD)	440-448	I70-I79
Stroke	*Prior to 30 June 1995*: 430, 431, 432.9, 434, 436	I60, I61, I62.9, I63, I64
	1 July 1995 - 30 June 1999: 430, 431, 432.9, 433.x1, 434.x1, 436	
Chronic kidney disease (CKD)	*Prior to 31 Dec 1987*: 250.3, 590.0, 590.2, 590.3, 590.8, 593.0, 593.1, 593.2, 593.6, 593.8, 593.9, 599.7, 753.0, 753.1, 753.2, 753.4, 996.1, V42.0, V45.1, 403, 404, 405, 580, 581, 582, 583, 585, 586, 587, 588, 589, V56	E10.2, E11.2, E12.2, E13.2, E14.2, I15.0, I15.1, N39.1, N39.2, T82.4, Z94.0, Z99.2, I12, I13, N00, N01, N02, N03, N04, N05, N06, N07, N08, N11, N12, N14, N15, N16, N18, N19, N25, N26, N27, N28, Q60, Q61, Q62, Q63, Z49
	1 Jan 1988 - 30 June 1995: 250.4, 590.0, 590.2, 590.3, 590.8, 593.0, 593.1, 593.2, 593.6, 593.9, 599.7, 753.0, 753.1, 753.2, 753.4, 996.1, V42.0, V45.1, 403, 404, 580, 581, 582, 583, 585, 586, 587, 588, 589, V56, 405.01, 405.11, 405.91, 405.02, 405.12, 405.92, 593.81, 996.81	
	1 July 1995 - 30 June 1999: 250.4, 590.0, 590.2, 590.3, 590.8, 593.0, 593.1, 593.2, 593.6, 593.9, 599.7, 753.0, 753.1, 753.2, 753.4, 996.1, V42.0, V45.1, 403, 404, 580, 581, 582, 583, 585, 586, 587, 588, 589, V56, 405.01, 405.11, 405.91, 405.02, 405.12, 405.92, 593.81, 996.81	
Diabetes	250	E10-E14

ICD: International Classification of Diseases; STEMI: ST-elevation myocardial infarction;
NSTEMI: Non-ST-Elevation Myocardial Infarction; CHD: Coronary heart disease; MI: Myocardial infarction

Appendix 6: Age and sex-standardised admission rates in a sensitivity analysis where equivocal classifications were included in the diagnostic category

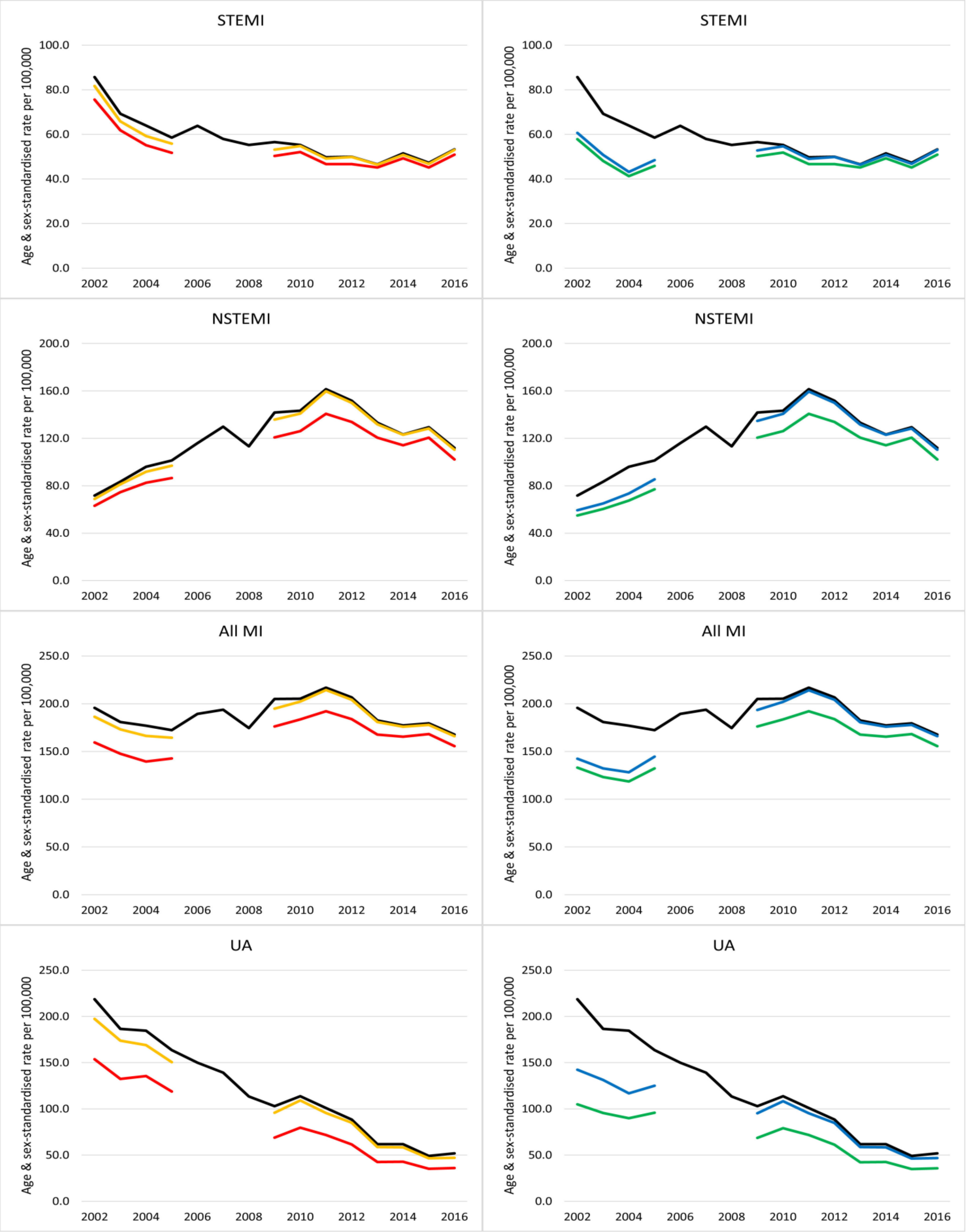

ICD-coded admissions
Admissions with any biomarker test
Diagnostic biomarker (non-diagnostic for UA)

ICD-coded admissions
Admissions with cTn/hs-cTn test
Diagnostic cTn/hs-cTn (non-diagnostic for UA)

STEMI: ST-elevation myocardial infarction, NSTEMI: Non-ST-Elevation Myocardial Infarction, MI: Myocardial infarction, UA: Unstable angina. All MI refers to STEMI, NTEMI, or unspecified MI

Appendix 7: Age-standardised admission rates by biomarker status among male patients

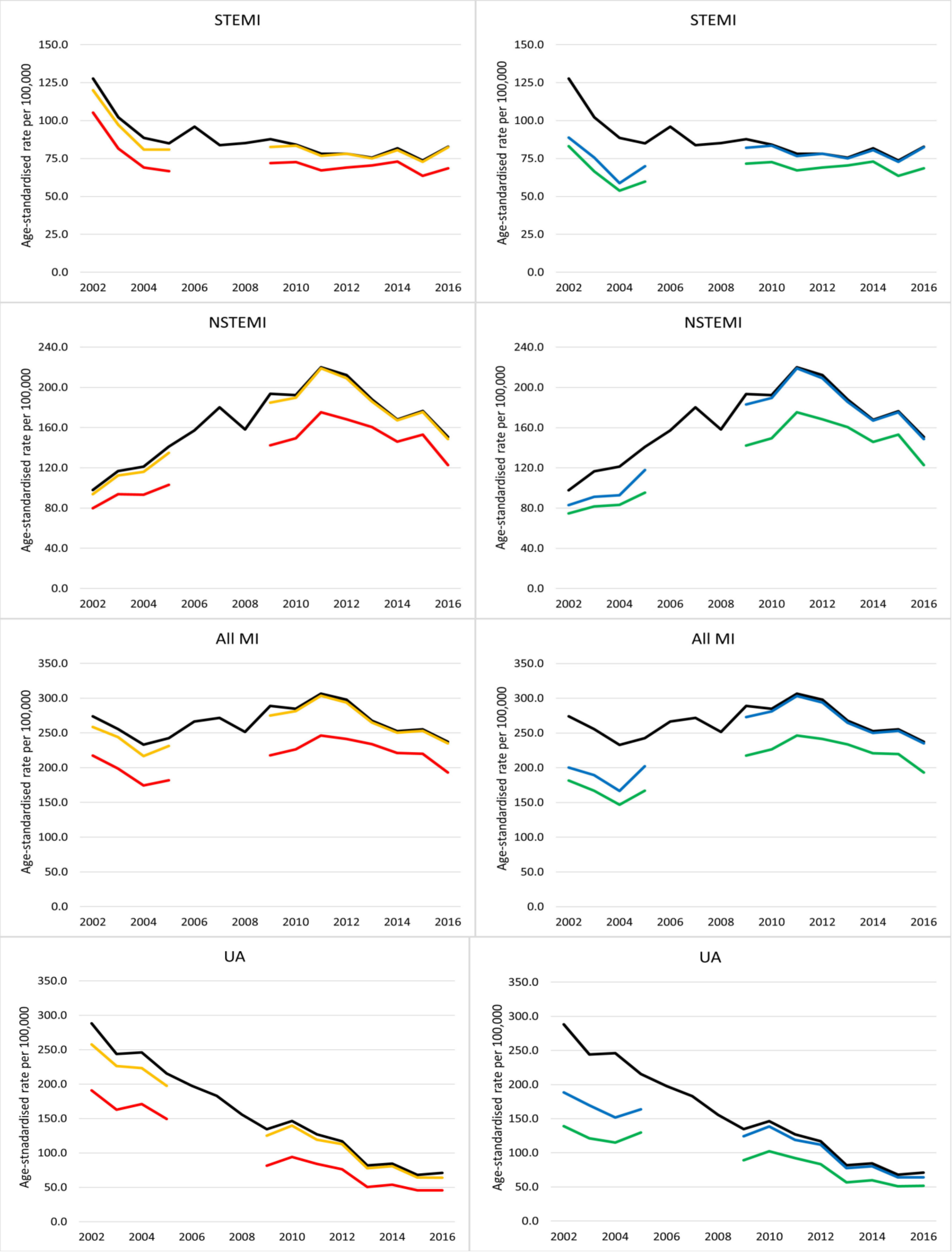

ICD-coded admissions
Admissions with any biomarker test
Diagnostic biomarker (non-diagnostic for UA)

ICD-coded admissions
Admissions with cTn/hs-cTn test
Diagnostic cTn/hs-cTn (non-diagnostic for UA)

STEMI: ST-elevation myocardial infarction, NSTEMI: Non-ST-Elevation Myocardial Infarction, MI: Myocardial infarction, UA: Unstable angina. All MI refers to STEMI, NTEMI, or unspecified MI

Appendix 8: Age-standardised admission rates by biomarker status among female patients

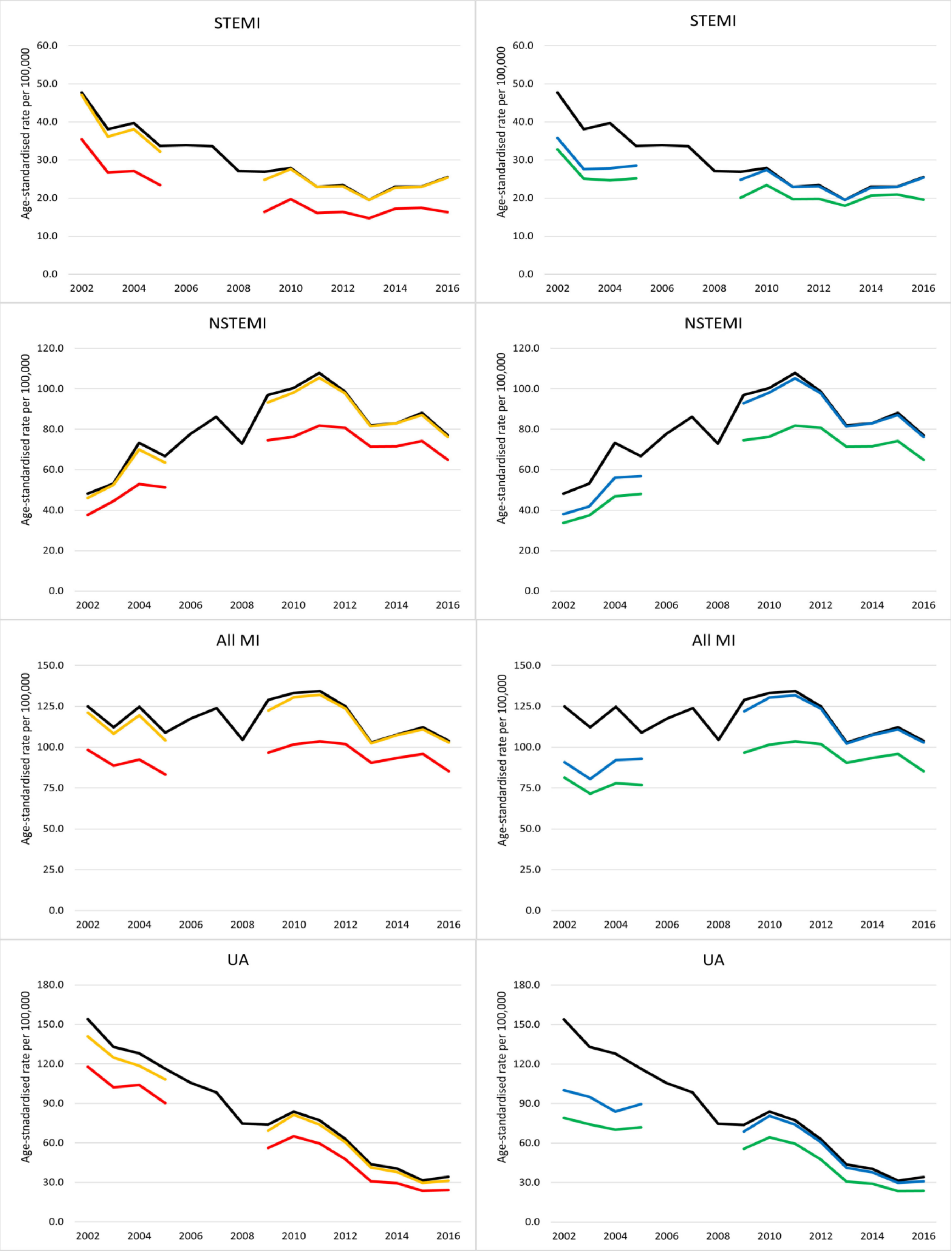

ICD-coded admissions
Admissions with any biomarker test
Diagnostic biomarker (non-diagnostic for UA)

ICD-coded admissions
Admissions with cTn/hs-cTn test
Diagnostic cTn/hs-cTn (non-diagnostic for UA)

STEMI: ST-elevation myocardial infarction, NSTEMI: Non-ST-Elevation Myocardial Infarction, MI: Myocardial infarction, UA: Unstable angina. All MI refers to STEMI, NTEMI, or unspecified MI

Appendix 9: ICD-coded age and sex-standardised admission rates in the whole of WA, and age-standardised rates by sex

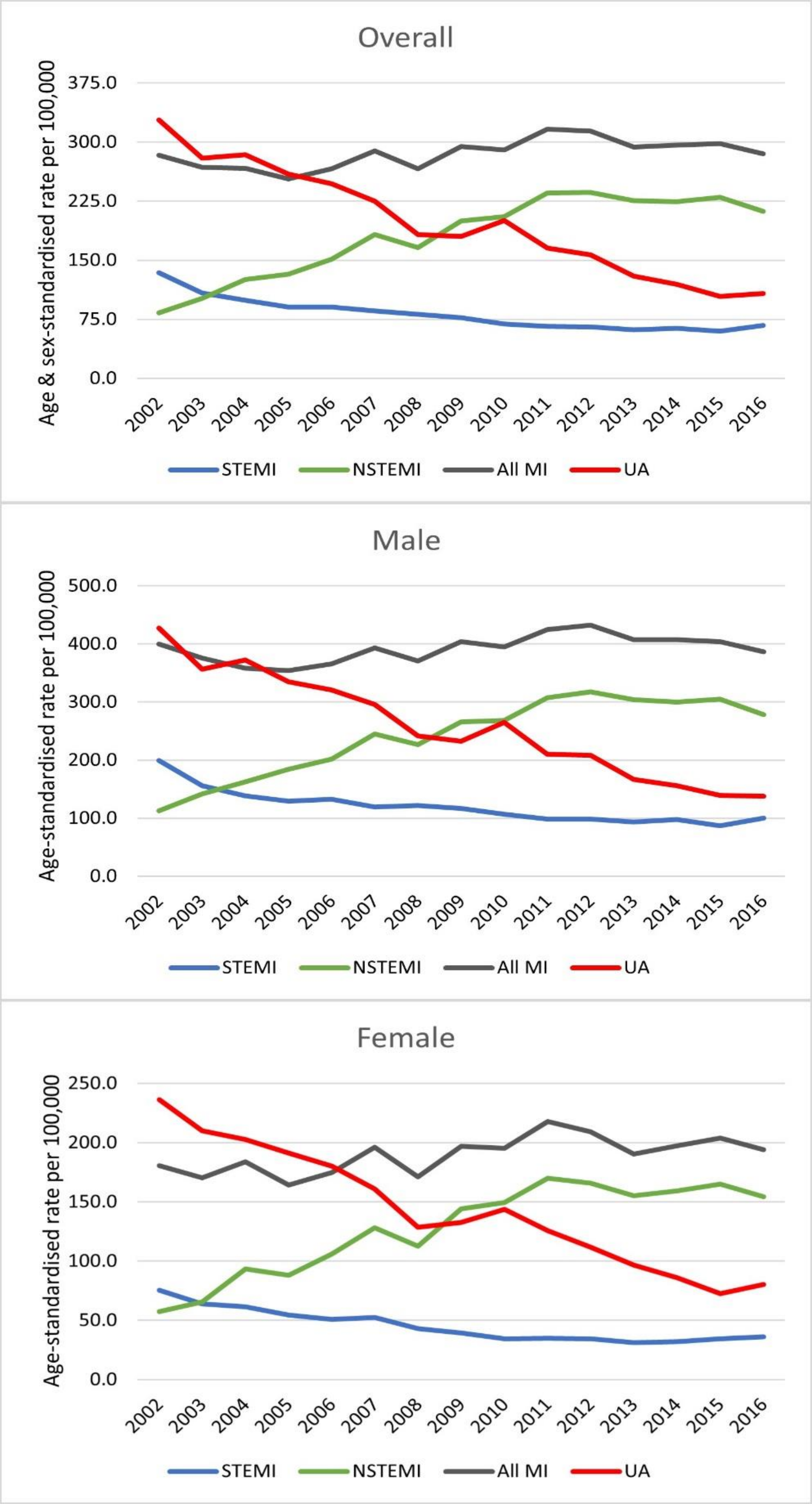

Appendix 10: Sex-specific temporal trends in admission rates for acute coronary syndromes by biomarker classification status in two periods

	Average annual % change (95% CI)							
	**20 to 54**		**55 to 74**		**75 and above**		**Overall**	
**Male**	**2002-2010***	**2011-2016**	**2002-2010***	**2011-2016**	**2002-2010***	**2011-2016**	**2002-2010***	**2011-2016**
**STEMI**								
ICD-coded	-1.5 (-3.0, 0.0)	-4.7 (-7.6, -1.7)	-5.7 (-6.9, -4.5)	1.5 (-0.9, 3.9)	-9.6 (-11.3, -7.8)	-0.3 (-4.1, 3.6)	-2.9 (-4.0, -1.7)	0.4 (-1.7, 2.6)
Diagnostic biomarker	1.6 (-0.8, 4.0)	-2.8 (-6.7, 1.2)	-1.7 (-3.7, 0.3)	0.0 (-3.2, 3.3)	-7.9 (-11.0, -4.8)	2.6 (-2.8, 8.4)	-1.8 (-3.2, -0.4)	-0.4 (-2.7, 1.9)
Diagnostic cTn/hs-cTn	5.0 (2.4, 7.7)	-2.8 (-6.7, 1.2)	1.7 (-0.4, 3.9)	0.0 (-3.2, 3.3)	-4.3 (-7.6, -0.8)	2.6 (-2.8, 8.4)	1.7 (0.2, 3.2)	-0.4 (-2.7, 1.9)
**NSTEMI**								
ICD-coded	10.6 (8.6, 12.6)	-3.2 (-5.5, -0.9)	8.3 (7.1, 9.6)	-2.0 (-3.5, -0.6)	11.0 (9.6, 12.5)	-1.4 (-3.1, 0.3)	7.9 (6.8, 9.0)	-7.2 (-8.6, -5.8)
Diagnostic biomarker	7.1 (4.3, 10.1)	-6.6 (-9.9, -3.1)	6.4 (4.5, 8.4)	-6.6 (-8.8, -4.4)	9.4 (7.3, 11.6)	-5.2 (-7.8, -2.6)	7.7 (6.4, 8.9)	-6.1 (-7.6, -4.6)
Diagnostic cTn/hs-cTn	8.4 (5.4, 11.4)	-6.6 (-9.9, -3.1)	8.4 (6.4, 10.4)	-6.6 (-8.8, -4.4)	11.2 (9.0, 13.5)	-5.2 (-7.8, -2.6)	9.4 (8.1, 10.8)	-6.1 (-7.6, -4.6)
**All MI**								
ICD-coded	2.0 (0.9, 3.2)	-4.0 (-5.8, -2.2)	-0.2 (-1.0, 0.7)	-1.2 (-2.4, 0.1)	0.8 (-0.2, 1.8)	-1.8 (-3.3, -0.3)	1.6 (0.8, 2.3)	-5.1 (-6.2, -3.9)
Diagnostic biomarker	2.8 (1.2, 4.5)	-5.7 (-8.0, -3.3)	1.5 (0.4, 2.7)	-4.5 (-6.2, -2.8)	3.0 (1.5, 4.4)	-5.1 (-7.2, -3.0)	2.0 (1.1, 2.9)	-4.4 (-5.7, -3.2)
Diagnostic cTn/hs-cTn	5.8 (3.8, 7.7)	-5.1 (-7.6, -2.5)	4.4 (3.0, 5.9)	-4.4 (-6.2, -2.5)	4.6 (2.8, 6.3)	-4.0 (-6.3, -1.6)	4.8 (3.8, 5.8)	-4.4 (-5.7, -3.2)
**UA**								
ICD-coded	-6.0 (-7.3, -4.8)	-17.4 (-19.9, -14.8)	-6.8 (-7.6, -6.1)	-6.4 (-8.0, -4.6)	-6.7 (-7.7, -5.7)	-10.2 (-12.4, -8.0)	-8.6 (-9.4, -7.9)	-9.5 (-9.9, -9.1)
Non-diagnostic biomarker	-7.6 (-9.9, -5.3)	-17.5 (-21.9, -12.9)	-9.6 (-11.0, -8.2)	-8.6 (-11.6, -5.4)	-9.1 (-10.9, -7.2)	-14.6 (-18.5, -10.5)	-9.1 (-10.1, -8.1)	-12.1 (-14.3, -10.0)
Non-diagnostic cTn/hs-cTn	-2.3 (-5.0, 0.4)	-17.4 (-21.7, -12.8)	-4.5 (-6.0, -2.9)	-8.4 (-11.5, -5.3)	-3.6 (-5.8, -1.4)	-14.6 (-18.5, -10.4)	-3.8 (-5.0, -2.7)	-12.0 (-14.2, -9.9)
**Female**								
**STEMI**								
ICD-coded	-5.3 (-8.6, -1.9)	-1.5 (-8.2, 5.7)	-7.6 (-9.8, -5.5)	-0.2 (-4.8, 4.7)	-9.8 (-11.6, -8.0)	1.6 (-2.6, 6.1)	-6.6 (-8.3, -4.8)	2.1 (-1.8, 6.0)
Diagnostic biomarker	-1.4 (-7.1, 4.7)	-1.9 (-11.5, 8.7)	-4.0 (-7.5, -0.5)	4.6 (-2.0, 11.5)	-10.7 (-13.7, -7.7)	-1.9 (-7.8, 4.5)	-7.0 (-9.1, -4.9)	0.7 (-3.4, 4.9)
Diagnostic cTn/hs-cTn	1.4 (-4.7, 7.9)	-1.9 (-11.5, 8.7)	-1.1 (-4.8, 2.7)	4.6 (-2.0, 11.5)	-7.3 (-10.4, -4.0)	-1.9 (-7.8, 4.5)	-3.8 (-6.0, -1.5)	0.7 (-3.4, 4.9)
**NSTEMI**								
ICD-coded	15.0 (11.3, 18.8)	0.9 (-3.2, 5.1)	9.2 (7.3, 11.2)	0.3 (-2.0, 2.7)	11.7 (10.3, 13.3)	-2.2 (-4.0, -0.5)	8.5 (7.1, 9.9)	-5.7 (-7.5, -3.8)
Diagnostic biomarker	10.3 (4.8, 16.0)	-0.5 (-7.1, 6.5)	6.5 (3.7, 9.5)	-4.3 (-7.8, -0.6)	9.4 (7.4, 11.6)	-4.4 (-7.0, -1.7)	8.7 (7.0, 10.3)	-4.0 (-6.0, -1.9)
Diagnostic cTn/hs-cTn	12.5 (6.7, 18.6)	-0.5 (-7.1, 6.5)	8.6 (5.5, 11.7)	-4.3 (-7.8, -0.6)	11.6 (9.5, 13.9)	-4.4 (-7.0, -1.7)	10.8 (9.1, 12.5)	-4.0 (-6.0, -1.9)

**All MI**								
ICD-coded	3.6 (1.3, 6.0)	0.3 (-3.2, 3.9)	0.8 (-0.5, 2.2)	0.0 (-2.1, 2.1)	1.1 (0.1, 2.1)	-2.7 (-4.2, -1.1)	0.9 (-0.1, 2.0)	-4.5 (-6.1, -2.8)
Diagnostic biomarker	4.4 (1.1, 7.9)	-2.5 (-7.4, 2.7)	1.5 (-0.4, 3.5)	-3.6 (-6.5, -0.7)	2.2 (0.8, 3.6)	-5.0 (-7.1, -2.8)	1.1 (0.0, 2.3)	-3.2 (-5.0, -1.3)
Diagnostic cTn/hs-cTn	7.0 (2.9, 11.3)	-0.9 (-6.4, 4.9)	3.8 (1.6, 6.1)	-2.1 (-5.3, 1.1)	3.6 (2.0, 5.3)	-4.2 (-6.6, -1.8)	4.0 (2.8, 5.3)	-3.2 (-5.0, -1.3)
**UA**								
ICD-coded	-1.8 (-3.7, 0.3)	-10.9 (-14.6, -7.1)	-6.0 (-7.1, -4.9)	-10.0 (-12.3, -7.7)	-9.3 (-10.3, -8.3)	-10.1 (-12.5, -7.7)	-8.7 (-9.6, -7.7)	-9.9 (-10.4, -9.3)
Non-diagnostic biomarker	0.2 (-3.8, 4.5)	-21.8 (-28.2, -14.9)	-6.7 (-8.7, -4.8)	-20.2 (-24.1, -16.0)	-10.8 (-12.5, -9.1)	-15.4 (-19.2, -11.3)	-8.2 (-9.4, -7.0)	-18.2 (-20.8, -15.6)
Non-diagnostic cTn/hs-cTn	8.1 (3.2, 13.3)	-21.8 (-28.2, -14.9)	-1.2 (-3.5, 1.1)	-20.5 (-24.4, -16.3)	-6.6 (-8.4, -4.7)	-15.4 (-19.2, -11.4)	-3.3 (-4.7, -1.9)	-18.4 (-20.9, -15.7)

* Excludes 2006-08 for non-ICD-coded trends. STEMI, ST-elevation myocardial infarction; NSTEMI, Non-ST-elevation myocardial infarction; MI, myocardial infarction; UA, unstable angina. All MI include STEMI, NSTEMI and unspecified MI
